# Motor system alterations in retired former athletes: the role of aging and concussion history

**DOI:** 10.1186/1471-2377-13-109

**Published:** 2013-08-26

**Authors:** Louis De Beaumont, Sébastien Tremblay, Luke C Henry, Judes Poirier, Maryse Lassonde, Hugo Théoret

**Affiliations:** 1 Centre de recherche en neuropsychologie et cognition (CERNEC), Université de Montréal, Montréal, Canada; 2 CHU Sainte-Justine, Montréal, Canada; 3 Douglas Mental Health University Institute, Perry Hall, 6875 Blvd Lasalle, Verdun, Quebec H4H 1R3, Canada; 4 Department of Psychology, University of Montreal, CP 6128, Succ. Centre-Ville, Montreal, QC H3C 3J7, Canada

**Keywords:** Motor learning, Retired athletes, Concussion, Motor cortex, Magnetic resonance spectroscopy

## Abstract

**Background:**

Retired athletes with a history of sports concussions experience cognitive and motor declines with aging, and the risk of severe neurodegenerative conditions is magnified in this population. The present study investigated the effects of aging on motor system metabolism and function in former university-level athletes who sustained their last concussion several decades prior to testing.

**Methods:**

To test the hypothesis that age and remote concussions induce functional as well as metabolic alterations of the motor system, we used proton magnetic resonance spectroscopy to detect metabolic abnormalities in the primary motor cortex and the serial reaction time task (SRTT) to evaluate motor learning.

**Results:**

Our results indicate that motor learning is significantly reduced in former concussed athletes relative to controls. In addition, glutamate/H_2_O ratio in M1 was disproportionately reduced in concussed athletes with advancing age and was found to strongly correlate with motor learning impairments.

**Conclusion:**

Findings from this study provide evidence that the acquisition of a repeated motor sequence is compromised in the aging concussed brain and that its physiological underpinnings could implicate disproportionate reductions of M1 glutamate concentrations with advancing age.

## Background

Sports concussions are alarmingly prevalent and their long-term and cumulative effects increasingly recognized in the sports culture [1]. The demonstration of chronic alterations of brain mechanisms and functions after sports concussions [2-14] has brought increased awareness and concerns about the safety hazards associated with the practice of contact sports due to its inherent risks of sustaining sports concussions. Perhaps even more disquieting is the recent association between remote sports concussions and neurodegenerative diseases. For example, there is a fivefold increase in the prevalence of mild cognitive impairments (MCI) in retired professional athletes who sustained three or more concussions [15], a condition that converts at a rate of 10-20% annually into dementia [16]. Likewise, several cases of chronic traumatic encephalopathy (CTE) involving severe motor manifestations have been reported in athletes who sustained repetitive contact sport-related head injury [17-20]. Although more common to boxing and wrestling, contact sport-related CTE has also been found in retired football, soccer and ice hockey players [18-20]. A recent study provided the first pathological evidence that CTE might be associated with the development of a motor neuron disease [21], characterized by severe weakness, atrophy, spasticity, and fasciculations several years before death combined with extensive tau neurofibrillary changes, motor neuron loss, and corticospinal tract degeneration [22]. This is consistent with the known elevated incidence of amyotrophic lateral sclerosis (ALS) in varsity athletics [23], football [24] as well as professional soccer players [25]. At a subclinical level, otherwise healthy former athletes with remote concussions exhibit significant motor execution slowness (bradykinesia) relative to unconcussed counterparts [9]. These bradykinesia symptoms in former concussed athletes were found to be closely related to excessive intracortical inhibition of the primary motor cortex (M1), the latter finding also being reported in young concussed athletes [7,8,10]. Recently, asymptomatic concussed university-level football players who were tested more than nine months after their last concussion presented suppressed LTP/LTD-like plasticity (long-term potentiation and long-term depression) that strongly associated with reductions in motor sequence learning. The extent of LTP/LTD suppression was found to be directly related to lifelong M1 intracortical inhibitory dysfunction in the concussed brain.

This prompted us to examine how aging combined with a history of remote concussions interact to affect M1 metabolism and function in former university-level athletes. Knowing that both sports concussion [10] and age [26] alter motor sequence learning, we tested whether concussion and age would further impair motor sequence learning in former athletes who sustained their last concussions more than three decades ago relative to unconcussed former university-level athletes of equivalent age. Likewise, in light of recent evidence suggesting that age and concussions both reduce cortical NAA and glutamate levels [27-29], this study sought to determine whether neurometabolic abnormalities are observed in M1 of former concussed athletes, and if so, whether they relate to possibly reduced M1-dependent motor sequence learning.

## Methods

### Participants

All 30 participants included in this study were former university-level athletes between the ages of 51 and 75 recruited through university athletics organizations (refer to Table 1 for participants demographics). All participants were Caucasian males who played for their respective college or university hockey/football team. Participants were included if they met all of the following criteria: no history of alcohol abuse and/or substance abuse; no medical condition requiring daily medication or radiotherapy (malignant cancers, diabetes, hypertension and/or other cardiovascular diseases); no previous history of psychiatric illness, learning disability, neurological disorder (seizure or brain tumour) or TBI unrelated to contact sports. T2 MRI images were collected for diagnostic purposes and read by a neuroradiologist blinded to group classification. No anatomical anomalies were detected in any participant. Participants included in the present study were all right-handed and had no history of concussion after their university years. To better control for data contamination due to the protective properties of regular physical activity on the development of Alzheimer’s disease (Lindsay et al., 2002), participants had to engage in 1-hour physical activity session, such as playing recreational non-contact hockey and/or football, tennis, golf, hiking, skiing or taking walks, at least three times weekly at the time of testing. The nature of physical activities that participants engaged in was comparable in both experimental groups. Two participants were excluded because they could not recollect sufficient information about their concussion history to enable group classification. Finally, performance at the Mini-Mental Status Examination (MMSE) had to be ≥ 27 out of 30 for participants to take part in this study. The MMSE cutoff score was set particularly high as high cutoffs allow greater sensitivity to cognitive impairment [30], especially in highly educated participants [31], to avoid potential contamination from early cognitive impairment.

**Table 1:** Participants demographics and concussion history information

	**Former University-level athletes**	
	**Concussed (n = 15)**	**Controls (n = 15)**
Age at recruitment (yrs), mean (SD)	60.87 (7.51)	58.13 (5.28)
Level of Education, mean ± SD	16.67 (4.07)	17.27 (3.45)
Mean number concussions sustained	2.07 (1.22)	-
Mean time since last concussion	37.07 (7.93)	-
Mean concussion severity	2.19 (1.06)	
Age range frequency (50–56)	6	5
Age range frequency (57–63)	5	5
Age range frequency (63+)	4	5
Apoϵ4 allele frequency	2	2

Abbreviations: *yrs* years; *Apoϵ* Apolipoprotein E; *SD* standard deviation.

Participants concussion history information and demographics by experimental groups. Age range frequency refers to participants count stratified by age range. Apoϵ4 allele frequency refers to Apoϵ4 allele carriers count by experimental groups. Mean concussion severity refers to the group average of the most severe concussion sustained for each concussed athlete.

Participants were divided into two groups. The experimental group consisted of 15 former university-level athletes with a mean age of 60.87 years (SD 7.51) and a mean level of education of 16.67 years (SD 4.07) who sustained their last sports concussion in early adulthood. Concussion history information was collected by a certified neuropsychologist. The number of concussions sustained ranged from 1 to 5 and the time elapsed since the last concussion spanned from 29 to 53 years (mean 37.08 SD 7.10). The severity of concussions sustained in former athletes ranged from Grade 1 (concussion symptoms or mental status abnormalities on examination that lasted for less than 15 minutes, no loss of consciousness (LOC)) to Grade 3 (LOC, either brief (seconds) or prolonged (minutes)) according to the American Academy of Neurology practice parameters [32]; they all classified as mild traumatic brain injury on the Glasgow Coma Scale (scoring between 13 to 15).

The control group included 15 former university-level athletes with a mean age of 58.13 (SD 5.28) and a mean level of education of 17.27 (SD 3.45) who reported no prior history of concussion or neurological insult. The two groups did not differ according to age (t_*1, 28*_ = 1.15, *p* = .259) or level of education (t_*1, 28*_ = 0.44, p = .666). The study was approved by the University of Montreal ethics committees and all participants provided written informed consent prior to testing. This study was performed with the ethical standards laid down in the 1964 Declaration of Helsinky. Participants received a financial compensation of $60 CDN for their participation.

### Procedure

Participants underwent a single testing session including the administration of the concussion history questionnaire, the general health questionnaire (refer to the following reference for a more detailed description [9]), the motor sequence learning task, magnetic resonance spectroscopy and saliva sample collection.

#### General health questionnaire

A semi-structured health questionnaire was administered to screen for pre-determined inclusion criteria about lifestyle characteristics, life events and medical conditions that are known to exert an influence on general brain function. More specifically, the assessment of lifestyle and life habits included open and more structured questions about physical and cognitive activities engaged in as well as a history of substance abuse. This general health questionnaire also inquired about cardiovascular, neurological and psychiatric illnesses experienced during and after the university years as well as daily medications or treatment therapies that are known to exert an impact on brain function. Participants were also asked whether they suffered from chronic medical conditions altering motor system functions. Lastly, former athletes were asked to report recent subjective changes with their memory and other issues related to changes in cognition.

#### Serial reaction-time task (SRTT)

**Figure 1:**
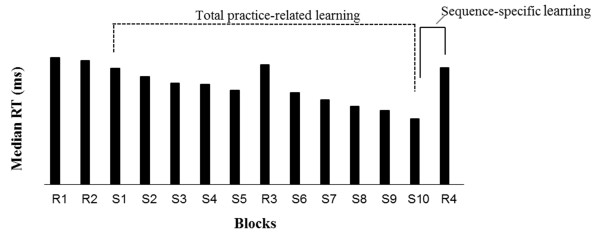
Representative serial reaction time task (SRTT) design.

The SRTT used in this study was identical to that previously used with young concussed athletes tested in our laboratory [10]. Participants were seated on a straight back chair with elbows flexed at an angle of 90°. They performed a modified SRTT [33] running on SuperLab (version 4.0; Cedrus, San Pedro, CA). The GO signal was displayed on the computer screen and consisted of one asterisk and three dots evenly spaced on an invisible horizontal plane, all appearing simultaneously. The position of the asterisk varied across trials among the four possible locations and indicated the required key press [33]. Participants were instructed to respond as fast and accurately as possible to the position of the asterisk by pressing the corresponding key with the predetermined finger (index finger for key 1, middle finger for key 2, ring finger for key 3, and little finger for key 4). A correct key press was required for the next trial to appear on the computer screen. Response time was defined as the time interval between stimulus presentation and the correct key press. Participants performed a total of 14 blocks separated by pauses and each block consisted of 10 presentations of the same 12-item sequence for a total of 120 key presses per block. They were instructed to perform the task with their dominant hand and to keep the appropriate finger on each predetermined key at all times. The two initial blocks consisted of stimuli presented in random order (random blocks) that differed from the predetermined repeating sequence. The first two random blocks (R1 and R2) were provided for participants to get familiar with the task. Blocks 3 to 7 and 9 to 13 corresponded to training blocks during which participants were presented with the following predetermined, repeating 12-item sequence (Sequence (S) : 4–2–3–1–1–3–2–1–3– 4–2– 4). Learning blocks were named according to their respective order preceded by the letter *S*. Sequence-specific learning was computed as the difference in median response time between the last sequence block (S10) and the last random block (R4) [34]. Total practice-related learning was calculated as the median response time difference between the first sequence block (S1) and the last sequence block (S10). Refer to Figure 1 for a graphical presentation of the SRTT paradigm.

#### Magnetic resonance spectroscopy (H-MRS)

**Figure 2:**
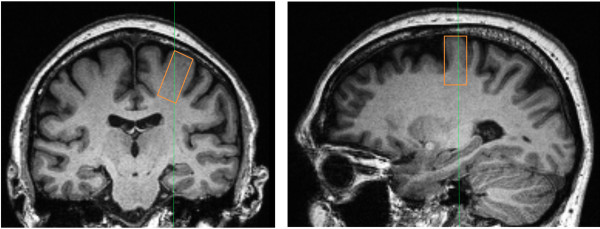
Region of interest for H-MRS examination.

All MR examinations were performed on a Siemens 3 T Magnetom TIM TRIO scanner with a 12-channel head coil (Siemens, Erlangen, Germany). 3D high resolution T1-weighted images of the brain were acquired using a sagittal MP-RAGE sequence (TR = 2300 ms; TE = 2,91 ms; Slices = 176) with a 1 mm^3^ resolution. T2-weighted images were obtained using a turbo spin-echo sequence (TR = 3000 ms; TE = 78 ms; Slices = 48) for diagnostic purposes. Proton magnetic spectra (^1^H-MRS) were obtained from voxels (voxel size of 16 mm × 20 mm × 32 mm) localized over the hand representation of the left primary motor cortex via high resolution T1 images using the anatomical landmarks proposed by Yousry and colleagues [35] (Figure 2). The position of a fixed-dimension virtual acquisition box was individually adjusted over the ROI in order to maximize the amount of gray matter included. All voxels contained a mixture of grey and white matter while investigators and MRI technicians conjointly performed online monitoring of potential signal artefacts from ventricles, fatty tissues and bones. Investigators as well as MRI technicians closely monitor signal artefact rejection Proton signal detection using the point-resolved spectroscopy pulse sequence (PRESS) was performed after suppression of the water signal with the chemical shift selective sequence. Consistent with a previous H-MRS study from our group conducted with an aging population [36], H_2_O signal was acquired for internal reference using a PRESS sequence with unsuppressed water signal [37]. Acquisition parameters were the following: TR = 1200 ms; TE = 30 ms; 128 averages. Free induction decays were transferred to a Silicon Graphics workstation and processed with the LCModel software version 6.1[38]. N-Acetylaspartate (NAA), glutamate and H_2_O were quantified.

#### DNA extraction

DNA extraction from saliva samples was performed using Oragene OG-250 s kits (DNA Genotek, Ottawa, Canada) and participants were genotyped for APOE 112 (rs429358)-158 (rs7412) polymorphisms. We carried out polymerase chain reaction (PCR) amplification as described previously [39]. APOE polymorphisms were subsequently determined via an established pyrosequencing protocol [39] with the following oligo sequencing (**APOE 158:** 5′**-**CCGATGACCTGCAGA-3′). Sequences to analyze were GT/CGCGGCCGC and AGT/CGCCTG for the multiplex APOE 112–158 polymorphisms.

#### Statistical analysis

All values are expressed as means (SD). Data were analyzed with SPSS 16 (SPSS, Chicago, IL). Significance was defined as *p* < .05, bilaterally. Effect sizes for mean differences are estimated with *partial eta squared.* Motor sequence learning ratios at the SRTT task and H-MRS data were subjected to between-group ANCOVAs with age, level of education and APOE genotype as covariates. Two-tailed Pearson correlations, corrected for multiple comparisons with False Discovery Rate (FDR), were computed between SRTT and H-MRS data that significantly discriminated groups. The combined effects of concussions with age on M1 metabolite concentration ratios were explored using standard two-way ANOVA models.

## Results

### Serial reaction time task

**Figure 3:**
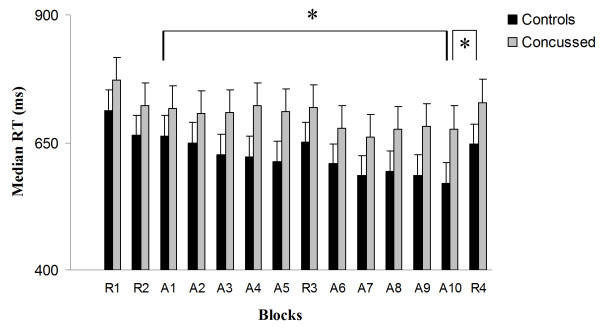
Response time in random and sequence blocks of the SRTT across groups.

Data for one concussed athlete had to be excluded from further analyses as the participant did not keep his fingers on predetermined press buttons at all times. The Training block effect was significant when percent change in median reaction times ((S1-S10)/S1) for each participant was used to compute a between-group ANCOVA with age, level of education and APOE genotype as covariates (*F*_1, 29_ = 4.342; *P* = .048; partial eta squared (η_p_^2^) = 0.164*;* Figure 3). This indicates that former concussed athletes improved significantly less than controls after 10 training blocks of the repeating 12-item sequence. The *sequence-specific* effect of learning was also significant when percent change in median reaction times ((S10-R4)/S10) for each participant was used to compute a between-group ANCOVA with age, level of education and APOE genotype as covariates (*F*_1, 29_ = 4.906; *P* = .036; partial eta squared (η_p_^2^) = 0.164*;* Figure 3). This indicates that former concussed athletes benefited significantly less than controls from 10 training blocks of a repeating sequence relative to the subsequent random block.

The main effect of group on median reaction times from the Group X Block (all 10 training blocks) ANCOVA was not significant (*F*_1, 29_ = 2.396; *P* = .134; partial eta squared (η_p_^2^) = 0.087. Median reaction times across random blocks did not differ between groups (*F*_1, 29_ = 1.841, *P =* .187). There was no group difference in mean response accuracy in sequence blocks [concussion group: 88.2% ± 5.6; controls: 89.1% ± 4.5; (*F*_1, 29_ = 0.32; *P =* .471) or random blocks [concussion group: 86.8% ± 7.8; controls: 88.2% ± 6.9; (*F*_1, 29_ = 0.41, *P =* .426).

### Magnetic resonance spectroscopy

H-MRS data from two participants had to be excluded from further analyses due to technical difficulties during data acquisition. Prior to using H_2_O as a reference for other metabolites of interest, we found that mean absolute water concentration in the ROI was non-statistically different across groups (t_1,27_ = .255; *P* = .40).

**Figure 4:**
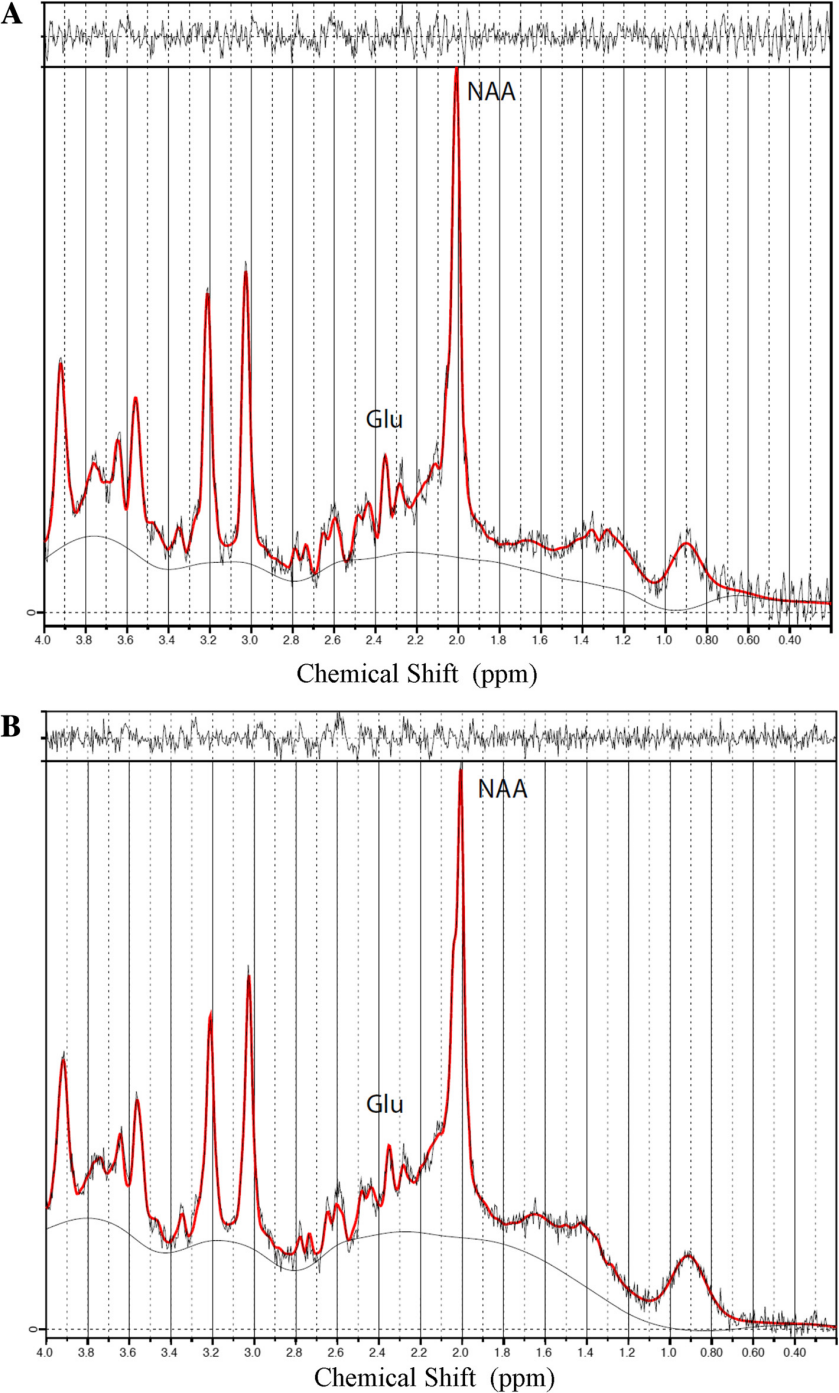
Water-suppressed representative H-MRS spectrum from each group (A- Control participant; B- Concussed participant).

H-MRS data collected in M1 revealed a near significant between-group effect on NAA/H_2_O (*F*_1, 27_ = 4.01; *P* = .057; η_p_^2^ = 0.143) [Mean NAA/H_2_O: (Control group: 1.61E-4 ± 1.63E-5; Concussion group: 1.85E-4 ± 3.37E-5)], but the ANCOVA with age, level of education and APOE genotype as covariates failed to reach significance. Glutamate concentration ratios with H_2_O as reference did not differ between groups (*F*_1, 27_ = .458; *P* = .505; η_p_^2^ = 0.018) [Mean Glutamate/H_2_O: (Control group: 1.24E-4 ± 1.97E-5; Concussion group: 1.19E-4 ± 1.74E-5)]. Since brain glutamate as well as NAA concentrations are known to reduce as a function of age [28], we tested whether the concussed brain experiences further glutamate/NAA concentration declines with advancing age. We found a significant Age * Group interaction for M1 glutamate concentration (*F*_1, 24_ = 4.616; *P* = .048; η_p_^2^ = 0.755; Figure 3), indicating that the effect of aging on M1 glutamate levels was significantly exacerbated in former concussed athletes. In contrast, the Age * Group interaction for NAA levels in M1 did not reach significance (*F*_1, 24_ = 3.50; *P* = .074; η_p_^2^ = 0.132). Figure 4 depicts representative, water-suppressed 1H-MRS spectra from each group.

**Figure 5:**
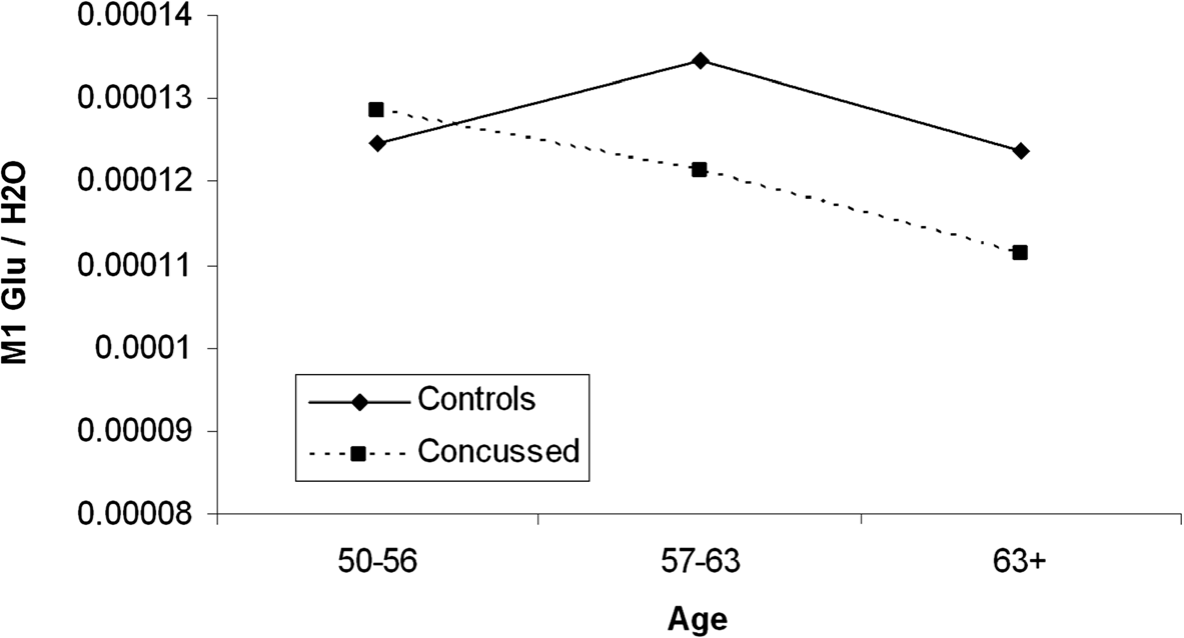
M1 Glutamate/H_2_O concentration ratio difference between former concussed athletes and controls with age.

Finally, the M1 glutamate/H_2_O ratio was strongly correlated with *sequence-specific learning* (R4 vs S10) in concussed athletes (r = 0.727; *P* = .007; Figure 5), while a similar association with *training block effects* (S10 vs S1) (r = 0.016; *P* = .962) was not significant.

### Correlational analyses with concussion history information

**Figure 6:**
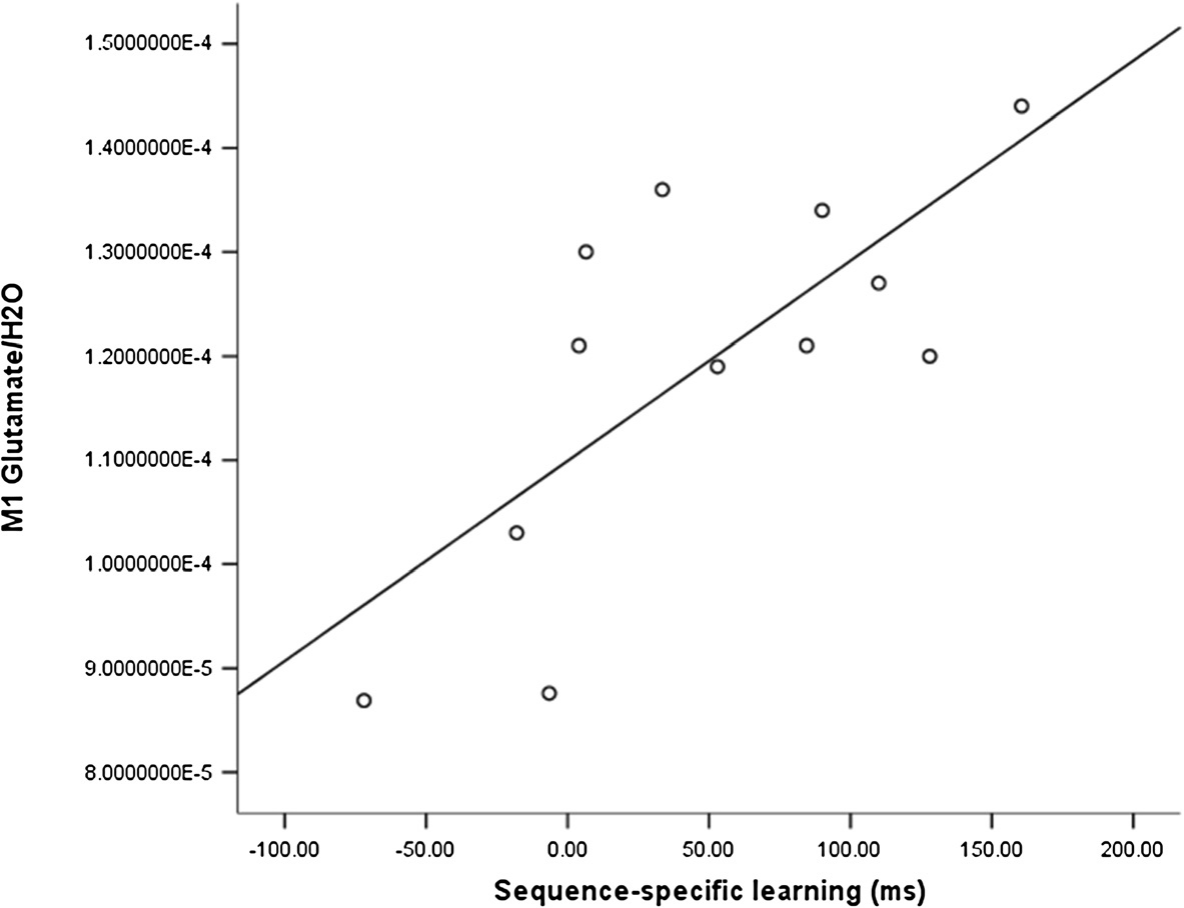
Correlation M1 glutamate and sequence-specific learning in concussion group.

Among concussed athletes, we found that the number of concussions sustained was negatively correlated with relative M1 glutamate/H_2_O levels (r = −.631; *P* = .021) as well as sequence-specific learning (r = −.597; *P* = .029) (ref to Figure 6). In contrast, neither concussion severity index nor the time elapsed since the last concussion significantly correlated with either relative M1 glutamate/H_2_O levels [Concussion severity (r = −.231; *P* > .05); Time since last concussion (r = −.179; *P* > .05)] or sequence-specific learning [Concussion severity (r = −.168; *P* > .05); Time since last concussion (r = −.211; *P* > .05)].

## Discussion

This study shows that the acquisition of a repeated sequence is significantly reduced in former university-level athletes with a reported history of sports concussion sustained more than three decades ago relative to unconcussed counterparts with equivalent demographics. In addition, glutamate/H_2_O ratio in M1 was found to be disproportionately reduced in former concussed athletes as they age, a finding that strongly correlated with decrements on M1-dependent motor sequence learning. Finally, strong negative correlations were drawn between the number of concussions sustained and both sequence-specific learning and relative M1 glutamate/H_2_O levels.

The main finding of this study is the alteration of motor sequence learning in former university-level athletes with a remote history of sports concussions. These data are in keeping with those collected at the same task in asymptomatic, active university-level athletes who were concussed more than nine months prior to testing [10]. This suggests that motor sequence learning impairments may be an early and long-lasting manifestation of concussive injury in former athletes who sustained sports concussions in early adulthood. Abnormal motor sequence learning has been linked to suppressed LTP/LTD-like plasticity in M1 of active concussed university-level football players that were asymptomatic at the time of testing [10]. The extent of M1 LTP/LTD suppression was found to be directly related to M1 intracortical inhibitory dysfunction [10]. Importantly, a previous study confirmed the chronicity of M1 intracortical inhibition impairments in a sample of former university-level concussed athletes comparable to those recruited in the present study [9]. Taken together, these data suggest that chronic impairments of motor sequence learning found in former concussed athletes could be linked to altered M1 intracortical inhibition mechanisms possibly via compromised LTP/LTD synaptic plasticity in M1.

Equally important is the strong relationship found in former concussed athletes between impaired motor learning and reductions in glutamate concentration within M1. A previous H-MRS study conducted with normal subjects aged between 24 and 68 years demonstrated that older subjects had lower glutamate concentrations in the motor cortex compared to younger participants [28]. This finding corroborated histological evidence of increased deficits of the glutamatergic systems in the aging animal brain [40-42]. Since most of glutamate is found in neurons, with extracellular concentrations being relatively low under normal conditions due to its excitotoxic properties [43], declining glutamate concentration levels with aging is therefore expected to alter neuronal metabolism and function over affected brain regions [28]. Glutamate being heavily involved in functions such as motor behavior and cognition [44,45], high intracellular glutamate concentration levels are consistently associated with better performance on cognitive as well as motor learning tasks [46]. The association between altered motor sequence learning and M1 glutamate concentration reductions in former concussed athletes from this study provide further evidence for the crucial role of intracellular glutamate in motor learning.

In parallel, knowing that glutamate/H_2_O concentration ratios did not significantly differ by remote concussion history alone in former athletes, the present findings suggest that advancing age potentiates latent changes of M1 metabolism after sports concussions. Interestingly, a previous H-MRS study looking at neurometabolic changes in young university-level athletes in the acute post-concussion phase found reduced glutamate levels in M1 that related significantly with self-reported symptom severity [27]. A follow-up study later revealed, however, that these acute glutamatergic system anomalies found in M1 return to normal within 6 months of the injury [47]. Findings from this study suggest that H-MRS can only detect post-acute M1 glutamate concentration abnormalities after sports concussion when athletes reach a more advanced age.

Another secondary finding from this study shows that among concussed athletes, the number of remote concussions negatively correlates with both sequence-specific learning and relative M1 glutamate/H_2_O levels, such that former athletes who presented with a history of more concussions were those who had lowest relative M1 glutamate concentration levels and who showed least learning effects at the SRTT task. In contrast, neither the time elapsed since the last concussion nor concussion severity were found to significantly correlate with reduced glutamate levels or learning effects found in concussed athletes. Although only fragmentary considering the limited sample size and the retrospective nature of concussion self-reports, these correlational findings provide further support for the cumulative deleterious effects of concussion on brain dysfunction.

The self-reported concussion history is indeed a major limitation inherent to these studies on the remote effects of concussions as blows to the head without loss of consciousness were medically overlooked several decades ago. As described previously [9], participants who were uncertain about their answers to the concussion history form were excluded from further analyses. This precautionary step to restrict data contamination coupled with the very stringent set of exclusion criteria, however, limit generalizability of our findings and calls for further replications of the present study with a broader sample of former athletes that present with more diverse sports concussions history characteristics.

## Conclusion

In conclusion, findings from this study provide evidence that the acquisition of a repeated motor sequence is compromised in former concussed athletes and that physiological underpinnings could implicate disproportionate reductions of M1 glutamate concentrations with advancing age. Longitudinal follow-ups could be useful to explore how glutamate metabolism in M1 might potentially be involved in the future development of more severe, debilitating symptoms as former athletes get older.

## Competing interest

The authors declare that they have no competing interest.

## Authors’ contributions

LDB: As lead investigator on the project, LDB took charge of data collection, data analysis and writing the research report. ST: Took charge of recruitment, data collection, data analysis. LH: Participated to data analysis and writing the research report. JP: Participated to data analysis, study design and he reviewed the research report. ML: Participated to study design, provided funding for the project, reviewed the research report, provided necessary assistance for recruitment. HT: As senior author on the project, HT was the primary supervisor of the project and contributed significantly to each step of the scientific project. All authors read and approved the final manuscript.

## Pre-publication history

The pre-publication history for this paper can be accessed here:

http://www.biomedcentral.com/1471-2377/13/109/prepub
